# 

**Published:** 1868-06

**Authors:** 


### CONTENTS.

ORIGINAL COMMUNICATIONS.

Showing the Non-existence of two changes of Color of the Blood.......... 223

PROCEEDINGS OF SOCIETIES.

Minutes of the Ohio State Dental Society.......... 246

SELECTIONS.

Case of Reproduction of Tooth after gun-shot wound of Maxillary Bone.......... 249
Iodine and Carbolic Acid.......... 250
Impure Glycerine.......... 251
Aluminum *v.* Vulcanite. An Improvement in Artificial Teeth.......... 251
Pleasant Thoughts for Tobacco Users.......... 252
Bdellatomy. Taking Advantage of a Leech.......... 254
Sir James Paget on Over-feeding.......... 254
On Hæmorrhage from Waxy or Amyloid Degeneration.......... 255
Iowa State Dental Society.......... 256

EDITORIAL.

Gold Foil.......... 257
The Canada Journal of Dental Science.......... 258
Correction.......... 258
Chemical Food.......... 259
Proved by its Exceptions.......... 260
American Dental Association.......... 261
Friendly Suggestions.......... 262
A Law to Incorporate Dental Societies for the purpose of Improving and Regulating the Practice of Dentistry in the State of New York.......... 262
Brevities.......... 265

